# Commentary: Multipurpose prevention technologies—What about sexually transmitted infections?

**DOI:** 10.3389/frph.2023.1158528

**Published:** 2023-06-02

**Authors:** Anjanique Mariquit Rosete Lu, Lisa B. Haddad

**Affiliations:** ^1^ Department of Obstetrics and Gynecology, Northwell Health at South Shore University Hospital, Bay Shore, NY, United States; ^2^ Center for Biomedical Research, Population Council, New York, NY, United States

**Keywords:** sexually transmitted infections, multipurpose prevention technology, gonorrhea, chlamydia, syphilis

## Introduction

Over the past decade, developers have made advances in addressing sexual and reproductive health (SRH) needs through multipurpose prevention technologies (MPTs)—products designed to simultaneously prevent HIV, Sexually transmitted infections (STIs), and/or unintended pregnancy (1). Multiple studies have demonstrated users’ preference for methods that prevent pregnancy alongside HIV and/or STIs rather than a single indication (2–7). While there are limitations to interpreting results from hypothetical use studies, it is intuitive that individuals would prefer a product that offers multiple benefits. By leveraging contraceptive priorities, MPTs provide a potential solution to known challenges such as ongoing low uptake of HIV pre-exposure prophylaxis (PReP) (8) while reducing the stigma of prevention (5, 9–11).

**Figure 1:**
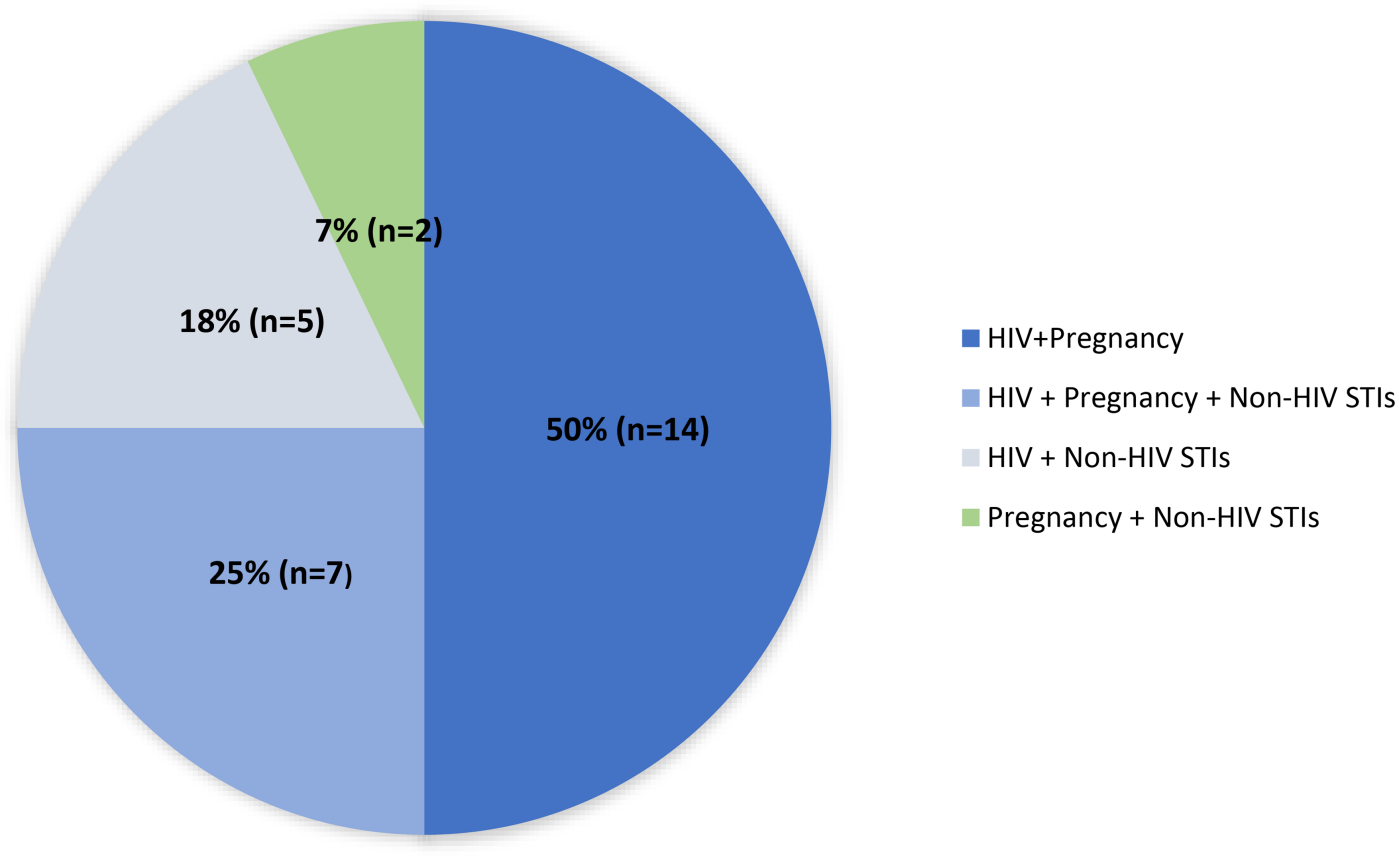
Multipurpose prevention technologies by indication (*n* = 28). Adapted from “MPT Pipeline by Indication Combination” by MPT 101, 2023 (https://mpts101.org/wp/wp-content/uploads/2023/01/MPT-Pipeline-Table_The-IMPT-Dec-2022_Indication-Combination.pdf). Copyright by IMPT for Reproductive Health. [Accessed January 29, 2023].

The majority of MPTs under development incorporate HIV prevention (Figure 1) (12) consistent with stakeholder prioritization and funding allocation. U.S. government research funding for HIV/AIDS totaled $1.4 billion dollars in 2018, dwarfing funding for all other STIs and contraception combined (Table 1) (13). This heavy focus on HIV has been appropriate given the large global costs and burden. Global HIV infection causes 47.63 million DALYs in 2019, compared to an estimated 8.58 billion global DALYs accounted by non-HIV STIs (14). With recent promising advances in HIV treatment and prevention, MPT initiatives and appropriate funding must now shift to advance more products preventing non-HIV STIs to curb the growing STI epidemic.

**Table 1:** Sexual and reproductive health research & development funding (US$ million) (2018).

Health issue	Funding (US$ million)
HIV/AIDS	1,442
STIs—excluding HPV	71
Contraception	64
Multipurpose prevention technologies	48

Source: G-finder global investment survey, 2020. Policy cures research. (https://gfinder.policycuresresearch.org/). [Accessed January 29, 2023].

## Why do we need to care about STIs?

Non-HIV STIs account for 98% of all prevalent STIs worldwide (15, 16). In 2016, the World Health Organization (WHO) estimated there were 376 million new infections of four curable STIs: chlamydia (127 million), gonorrhea (87 million), syphilis (6 million), and trichomoniasis (156 million) (15). Viral STIs such as genital herpes simplex virus (HSV) and human papilloma virus (HPV) also have notably been increasing in prevalence (15). In the U.S. alone, 2.4 million new cases of chlamydia, gonorrhea, and syphilis were reported in 2020 despite underreporting and reduced access to screening during the COVID pandemic (17). Despite global efforts to combat STIs, infections are at an all-time high and cost $2 billion in treatment annually (18, 19). Untreated, STIs have broad-reaching health impacts ranging from increased risk of HIV acquisition, cancer, chronic pelvic pain, infertility, preterm delivery, and neonatal morbidity and mortality (15). Many infections may be asymptomatic and go undiagnosed. Even when identified, treatment may be complicated by challenges such as medication shortages, emerging antibiotic resistance, and reinfection after inadequate partner treatment (20–23). Adverse health impacts and barriers to diagnosis and treatment make renewed dedication to STI prevention strategies critical.

Non-HIV STIs are inextricably linked to HIV and contraception. Infection with some STIs such as HSV, chlamydia and gonorrhea may increase the risk of HIV acquisition (24, 25). Use of certain contraceptives may be associated with increased acquisition of chlamydia and HSV (26, 27). While some of these findings may be linked to more frequent access of diagnostic services, biological plausible mechanisms for altered risk exist (28, 29). As the linkages expand beyond epidemiologic and behavioral sexual risk factors, developers have an enhanced imperative to develop overlapping prevention tools.

## Current and emerging tools for STI prevention

While not universally available, the global market for HIV prevention includes medications taken pre- or post-exposure with different formulations including daily pill, long-acting injectables and vaginal rings (30–32). Prevention for other STIs still largely relies on promoting healthy sexual behaviors and barrier method use—traditional approaches that have been inadequate in curbing rise in infections (33). The current STI prevention pipeline leans heavily on vaccine development. While there are only two vaccines currently on the market, both of which act against viral STIs (Hepatitis B and HPV) (21), several vaccines in development offer hope for broader protection. Several MPTs are integrating non-vaccine prevention methods, however these are mostly in the preclinical stages. Promising options currently being explored for STI prevention include the recently FDA approved on-demand vaginal pH modulator (VPM) Phexxi® (34) and doxycycline as a possible pre- or post-exposure bacterial STI prophylaxis (35, 36).

Triple protection MPTs with broad pregnancy, non-HIV STIs, and HIV prevention make up about a quarter of all MPTs in development (Figure 1) (12). Advancing triple-indication products would align with the overlapping risks faced by many people around the world (37) and simultaneously confront concerns of risk compensation. Risk compensation theorizes that individuals who use STI prevention methods might engage in high-risk sexual behavior such as condomless sex and increased number of sexual partners. Although it is unclear to what extent risk compensation occurs, this potential raises concern that use of a prevention method for one STI might contribute to increasing rates of other STIs (38). Monitoring MPTs’ impact on other STIs is critical as newer methods enter the market. As it is unlikely there will be one method that protects against all STIs, a diverse range of MPTs must be developed to enable individuals to prioritize STIs that are highly prevalent in their community.

## Discussion: a call to action

Efforts to counter the STI epidemic through MPTs rely on several key actions listed in Box 1. Exciting novel MPTs indicated for non-HIV STIs are already making their way through the development pipeline. Critical to success in the fight against STIs is momentum in R&D activities. This requires engaging researchers who are already focused on non-HIV STI projects as well as funders and impact investors who may be interested given their large potential market in developed countries, but who have not yet partnered with MPT developers. A key challenge to advancing these products is also ensuring that the regulatory environment facilitates approval of multipurpose prevention. The development of regulatory processes tailored specifically for the approval of MPTs will accelerate expansion of the STI prevention toolbox. With the traditional regulatory framework, multiple costly Phase 3 trials can be a barrier to advancing some indications, leading to the potential risk of secondary benefits not obtaining regulatory approval. As products advance, acknowledging that users value product characteristics other than effectiveness may open avenues for a broader array of options that address users’ needs. Ultimately, advancing MPTs indicated for non-HIV STIs will allow more individuals to achieve their sexual and reproductive health goals.

BOX 1 **Recommendations for future MPT product development.**

**Table:** 

**Resources**
• Continue to escalate investments for MPTs indicated for non-HIV STIs
• Engage legislators, policymakers, and investors who are interested in non-HIV STI prevention given the large potential market in developed countries, but who have not yet partnered with MPT development
• Seek non-traditional funding sources such as from impact investors and Corporate Social Responsibility
**Research**
• Expand STI prevention toolbox to diversify biomedical modalities beyond vaccines
• Strengthen research on current lagging topics such as nonviral STIs, antibiotic resistance and medication shortages
• Connect researchers working on non-HIV STI prevention with those working on contraception and/or HIV prevention
• Ensure MPTs under development represent a breadth of options to suit the needs of diverse users (e.g., with and without pregnancy prevention, hormonal and non-hormonal, ARV and non-ARV, on-demand, short acting, and long acting, user-controlled and provider initiated).
• Conduct studies to determine market size for non-HIV STI prevention in developed countries to support the business case for investment
**Regulation**
• Develop streamlined processes for regulatory approval of products with multiple benefits
• Ensure surveillance systems are in place to monitor the impact of new prevention technologies on STIs
